# Gut microbiota-derived metabolites in gastrointestinal cancer: immunomodulatory mechanisms via the tryptophan–AhR axis and translational perspectives

**DOI:** 10.3389/fimmu.2026.1862232

**Published:** 2026-07-09

**Authors:** Xue Xing, Hui Ying, Dong-Xia Wang, Shuang Han

**Affiliations:** 1 Department of Clinical Laboratory, The Second Affiliated Hospital of Dalian Medical University, Dalian, China; 2 College of Laboratory Medicine, Dalian Medical University, Dalian, China

**Keywords:** gastrointestinal cancer, gut microbiota, immune regulation, microbial metabolites, multi-omics integration, precision therapy, tryptophan–AhR axis, tumor microenvironment

## Abstract

The gut microbiota contributes to host physiological homeostasis through the production of diverse bioactive metabolites that orchestrate immune responses, maintain intestinal barrier integrity, and regulate metabolic signaling. Increasing evidence indicates that these microbiota-derived metabolites act as critical mediators linking microbial composition to gastrointestinal tumorigenesis. Among them, short-chain fatty acids (SCFAs), bile acids, and amino acid-derived metabolites—particularly tryptophan metabolites—have been most extensively studied. These metabolites influence tumor initiation and progression via multiple mechanisms, including modulation of inflammatory responses, epigenetic remodeling, control of cell proliferation and apoptosis, and reprogramming of the tumor microenvironment (TME). This review provides a systematic overview of the sources, biological functions, and mechanistic roles of major gut microbial metabolites in gastrointestinal cancer. We emphasize three interconnected regulatory axes: inflammation–immunity modulation, intestinal barrier homeostasis, and metabolism–epigenetic reprogramming, while highlighting the tryptophan–aryl hydrocarbon receptor (AhR) axis as a central immunoregulatory hub. SCFAs generally exert protective effects by reinforcing epithelial barrier function and supporting anti-tumor immunity, whereas bile acids display context-dependent duality, with secondary bile acids being closely associated with chronic inflammation, DNA damage, and colorectal cancer progression. Tryptophan metabolites primarily shape mucosal immunity and tumor immune escape through activation of AhR signaling. Despite substantial progress, important challenges remain, including incomplete causal understanding, marked inter-individual variability, and limited clinical translation. Future studies integrating multi-omics approaches, single-cell and spatial technologies, and prospective clinical validation will be essential for defining key metabolite networks and advancing metabolite-based biomarkers and precision therapeutic strategies.

## 1 Introduction

The gut microbiota constitutes a highly dynamic and metabolically active ecosystem composed of bacteria, fungi, viruses, and other microorganisms, and plays a central role in regulating host metabolism, maintaining immune homeostasis, and preserving intestinal barrier integrity (1–5). With the rapid development of high-throughput sequencing, metabolomics, and other multi-omics technologies, the complex interactions between the gut microbiota and host physiology have become increasingly well characterized. Among the diverse mechanisms linking the microbiota to disease, microbiota-derived metabolites have emerged as key functional mediators that translate microbial composition and activity into host inflammatory, immune, and metabolic responses (2, 4–9).

These metabolites are not merely byproducts of microbial metabolism; rather, they actively participate in host signaling networks. Through receptor-mediated pathways, transcriptional regulation, and epigenetic remodeling, they influence immune responses, cellular metabolism, and gene expression, thereby contributing to the initiation and progression of multiple diseases, including cancer (7, 10–14).

Gastrointestinal cancers encompass a heterogeneous group of malignancies, including colorectal cancer, gastric cancer, esophageal cancer, hepatocellular carcinoma, cholangiocarcinoma, and pancreatic ductal adenocarcinoma. These malignancies remain major contributors to cancer-related morbidity and mortality worldwide (15). Despite advances in screening, diagnosis, and treatment, the prognosis of many gastrointestinal malignancies remains unsatisfactory because of complex pathogenesis, marked intertumoral and intratumoral heterogeneity, and profound immune evasion. Therefore, clarifying the molecular mechanisms that drive tumor initiation and progression and identifying novel therapeutic targets remain urgent priorities.

Accumulating evidence has highlighted the essential role of gut microbiota-derived metabolites in gastrointestinal tumorigenesis (5, 7, 8, 10, 11, 16). Among the best-characterized metabolite classes are SCFAs, bile acids, and amino acid-derived metabolites, particularly tryptophan metabolites (10–12, 17–25). SCFAs generally exert anti-inflammatory and tumor-suppressive effects through activation of G protein-coupled receptors (GPCRs) and inhibition of histone deacetylases (HDACs) (13, 20, 23, 26). By contrast, bile acids influence tumor progression through nuclear receptor signaling, oxidative stress, and immune modulation (10, 21, 22, 27). Tryptophan metabolites contribute to immune tolerance and tumor immune escape primarily through activation of the AhR axis (12, 18, 19, 24, 28).

However, current knowledge remains fragmented. Many studies still focus on individual metabolites or isolated downstream pathways, even though the biological effects of microbial metabolites are highly dependent on metabolite abundance, ligand specificity, tumor stage, host metabolic status, and the local immune microenvironment (4, 5, 8, 11, 13, 22). In addition, much of the available evidence remains preclinical or associative, with limited causal validation and insufficient clinical translation (5, 27, 29). Therefore, this review provides a systematic and integrative overview of gut microbiota-derived metabolites in gastrointestinal cancer. We focus on three interconnected mechanistic axes—inflammation–immunity regulation, intestinal barrier homeostasis, and metabolism–epigenetic reprogramming—and further highlight the tryptophan–AhR axis as a central immunoregulatory hub. By constructing an integrated “metabolite–immune–tumor” framework, we aim to advance mechanistic understanding and support the development of novel diagnostic and therapeutic strategies (30–32).

To improve transparency, the literature discussed in this review was primarily identified through searches of PubMed, Web of Science, and Scopus databases. Priority was given to mechanistic studies, translational investigations, and high-impact reviews related to gut microbiota-derived metabolites, immune regulation, and gastrointestinal cancers. Particular emphasis was placed on studies investigating SCFAs, bile acids, tryptophan metabolites, and the tryptophan–AhR axis, as well as recent advances published through March 2026.

## 2 Mechanistic framework of gut microbiota-derived metabolites in gastrointestinal cancer

Gut microbiota-derived metabolites regulate gastrointestinal tumorigenesis through a complex and multilayered regulatory network rather than through isolated signaling pathways. Increasing evidence indicates that these metabolites act as key intermediates linking microbial composition and metabolic activity to host tumor biology by coordinating immune responses, epithelial homeostasis, metabolic reprogramming, and intracellular signaling cascades, thereby reshaping the TME (4, 5, 7, 8, 10, 11, 22, 28).

Recent studies have shifted the conceptual framework from single-metabolite effects toward a systems-level understanding of the “metabolite–immune–tumor” axis, emphasizing that microbial metabolites function as integrative regulators of immunometabolic networks rather than as independent effectors (4, 5, 8, 28).

Among the diverse microbial metabolites, SCFAs, bile acids, and amino acid-derived metabolites—particularly tryptophan metabolites—are the most extensively studied. These metabolites not only participate in host energy metabolism but also regulate tumor progression through receptor-mediated signaling, epigenetic remodeling, oxidative stress responses, and metabolic reprogramming (10–12, 17–24). Importantly, these metabolite classes do not act independently; instead, they exhibit extensive functional crosstalk, collectively shaping inflammatory responses, immune cell function, epithelial barrier integrity, and the TME, thereby forming an interconnected regulatory network (4, 5, 7, 8, 11, 22, 28).

**Figure 1:**
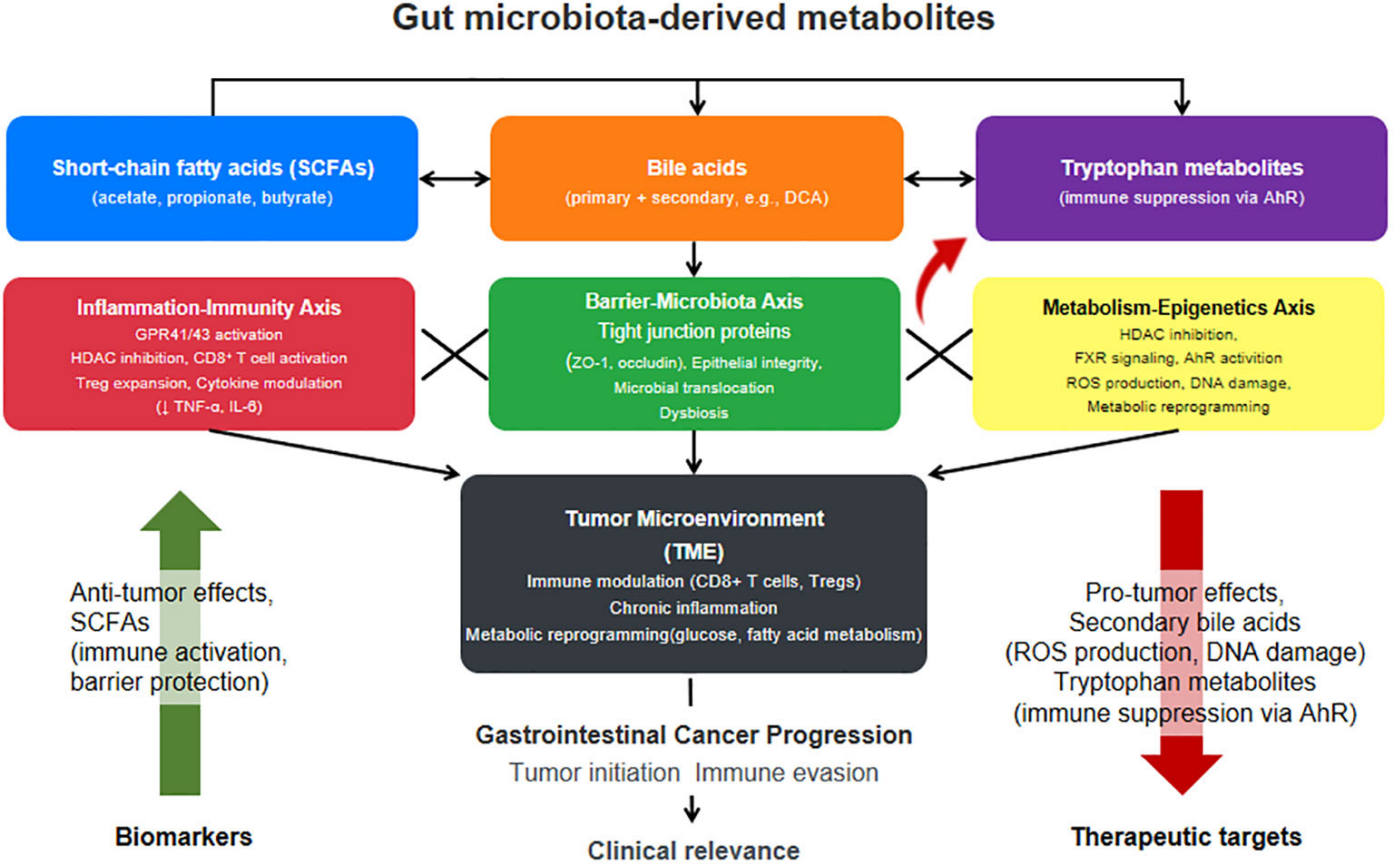
Mechanistic framework of gut microbial metabolites in gastrointestinal cancer. Conceptual framework linking microbial metabolites to gastrointestinal tumorigenesis through interconnected immune, barrier, and metabolic pathways. SCFAs generally support epithelial integrity and anti-tumor immunity, whereas dysregulated bile-acid and tryptophan-metabolite signaling may promote immune suppression and tumor progression.

**Figure 2:**
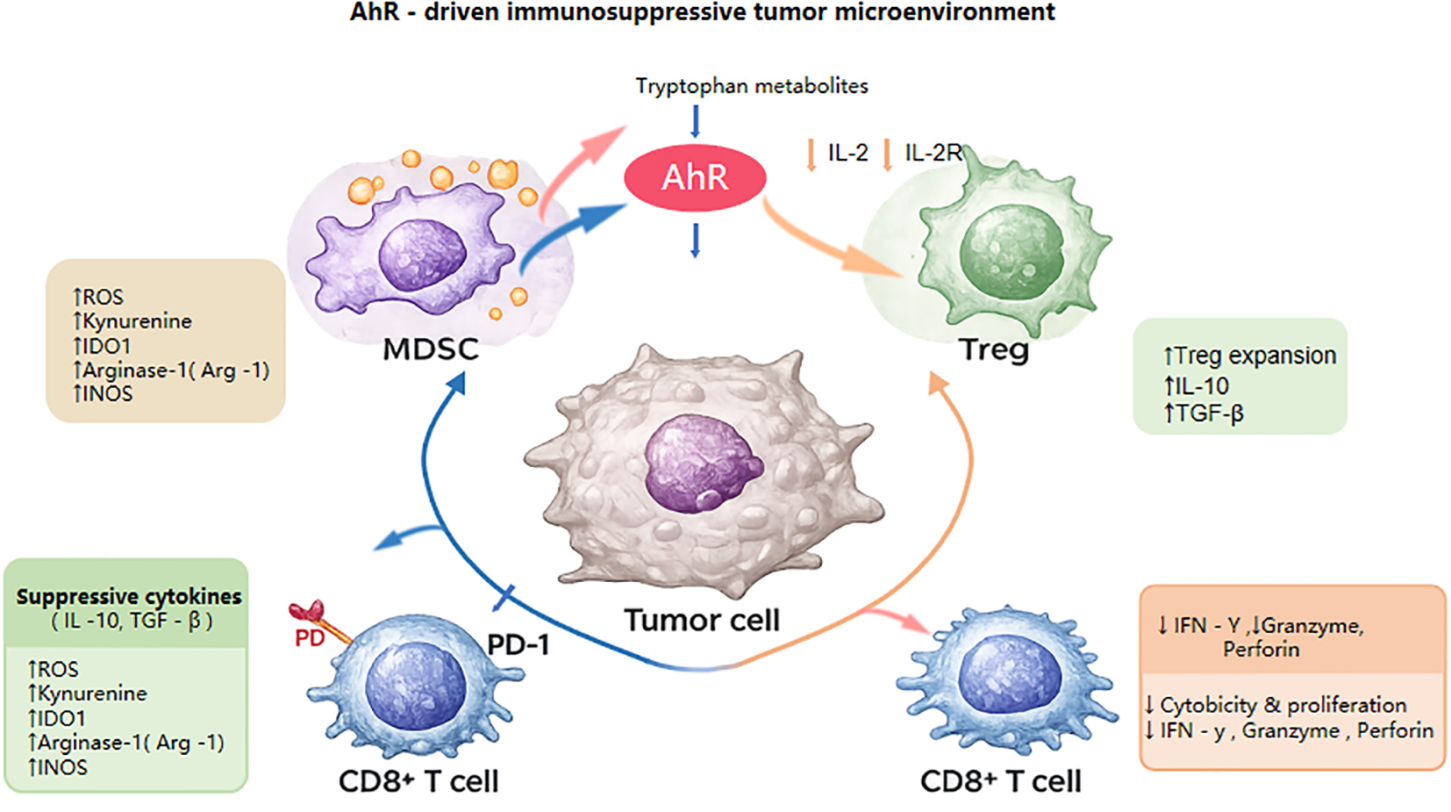
Integrated immunoregulatory network mediated by the tryptophan–AhR axis. Tryptophan metabolites, including kynurenine and microbiota-derived indole derivatives, engage the AhR in immune and tumor-associated cells to regulate immune tolerance, epithelial barrier integrity, and anti-tumor immunity. Kynurenine–AhR signaling promotes Tregs and MDSC expansion, suppresses CD8^+^ T-cell function, and supports an immunosuppressive TME, whereas indole–AhR signaling contributes to epithelial barrier maintenance, mucosal homeostasis, and balanced immune responses. Crosstalk with HIF-1α and NF-κB further integrates hypoxic, inflammatory, metabolic, and immune cues. The net immunological outcome depends on ligand source, pathway activation status, and local inflammatory conditions.

Based on current evidence, the mechanistic roles of gut microbiota-derived metabolites in gastrointestinal cancer can be broadly organized into three interconnected axes: inflammation–immunity regulation, barrier–microbiota interaction, and metabolism–epigenetic reprogramming (**Figure 1**). These axes are not independent; rather, they exhibit substantial crosstalk and together govern tumor initiation, progression, immune evasion, and therapeutic responsiveness (4, 5, 28) **Figure 2**.

### 2.1 Inflammation–immunity axis

Chronic inflammation is a major driver of gastrointestinal tumorigenesis, and gut microbiota-derived metabolites contribute to this process by modulating both innate and adaptive immune responses, thereby shaping inflammatory signaling, immune surveillance, and anti-tumor immunity (7, 12, 13, 20, 23).

Mechanistically, SCFAs enhance anti-tumor immunity primarily through activation of GPCRs, including FFAR3/GPR41, FFAR2/GPR43, and HCAR2/GPR109A, as well as through inhibition of HDACs. These actions promote CD8^+^ T-cell effector function, restrain excessive pro-inflammatory signaling, and modulate cytokine production partly through suppression of nuclear factor-kappa B (NF-κB) activation (13, 20, 23).

In contrast, secondary bile acids—particularly deoxycholic acid (DCA)—often exert immunosuppressive and tumor-promoting effects in pathological settings. Recent evidence indicates that microbiota-modified bile acids can impair CD8^+^ T-cell effector functions and promote immune tolerance, thereby facilitating colorectal cancer progression (21, 22, 27). In addition, bile acids can influence macrophage polarization and contribute to the establishment of tumor-associated macrophage (TAM)-like phenotypes, further reinforcing an immunosuppressive TME (11, 21, 22, 27).

Tryptophan metabolites represent another major immunoregulatory class and act largely through activation of the AhR. Activation of AhR promotes the expansion of regulatory T cells (Tregs) and myeloid-derived suppressor cells (MDSCs), both of which suppress anti-tumor immune responses and facilitate immune evasion (12, 18, 19, 24, 28). In addition, AhR signaling can impair dendritic cell maturation and antigen-presentation capacity (33), thereby weakening immune surveillance and further promoting tumor tolerance (19, 24, 25).

Collectively, these findings indicate that gut microbial metabolites orchestrate a dynamic balance between immune activation and immune suppression, thereby critically shaping tumor immunity.

### 2.2 Barrier–microbiota axis

The integrity of the intestinal barrier is essential for maintaining gut homeostasis and limiting the translocation of luminal microbes, microbial products, and inflammatory stimuli. Disruption of this barrier facilitates persistent immune activation, chronic inflammation, and tumor-promoting microenvironmental remodeling, thereby contributing to gastrointestinal tumorigenesis (2, 4, 5, 10, 20, 22).

SCFAs play a protective role in maintaining epithelial barrier function. They enhance the expression of tight junction proteins, including occludin, claudins, and zonula occludens-1 (ZO-1), promote epithelial cell proliferation and differentiation, and suppress excessive inflammatory signaling, thereby reducing intestinal permeability (34–39). In addition, SCFAs support epithelial regeneration and repair through adenosine monophosphate-activated protein kinase (AMPK)-dependent signaling, autophagy induction, and metabolic support for colonocytes (26, 38, 39).

In contrast, dysregulated bile acid metabolism can impair barrier integrity. Excess accumulation of secondary bile acids damages epithelial cells, perturbs tight junction organization, and increases intestinal permeability, thereby facilitating bacterial translocation and chronic inflammation (10, 21, 22, 40). Moreover, bile acids can reshape microbial composition and promote dysbiosis, which further amplifies epithelial dysfunction and inflammatory signaling (21, 22, 41, 42).

Emerging evidence suggests that microbial metabolites and the gut microbiota form a bidirectional regulatory loop that collectively establishes a “microbiota–metabolite–barrier” axis. Within this framework, alterations in microbial composition affect metabolite availability, whereas metabolite-dependent signaling in turn reshapes epithelial homeostasis and microbial ecology, ultimately influencing colorectal carcinogenesis (4, 5, 43).

### 2.3 Metabolism–epigenetics axis

Gut microbiota-derived metabolites also regulate gastrointestinal tumorigenesis through coordinated effects on host metabolism and epigenetic programming. These processes are increasingly recognized as core determinants of tumor initiation, progression, and therapeutic responsiveness, particularly through their influence on chromatin remodeling, oxidative stress, metabolic substrate utilization, and immunometabolic competition within the tumor microenvironment (4, 5, 8, 12, 18, 21, 26, 28).

SCFAs, especially butyrate, function as endogenous HDAC inhibitors and thereby alter chromatin accessibility and transcriptional programs. Through this mechanism, SCFAs can induce cell-cycle arrest, promote differentiation, and trigger apoptosis in tumor cells. In addition, SCFAs influence tumor-associated metabolism by modulating glycolytic activity, mitochondrial function, and nutrient utilization, thereby counteracting metabolic features associated with malignant progression (26, 44–47).

Bile acids contribute to tumorigenesis through metabolic and oxidative stress pathways. Secondary bile acids, particularly deoxycholic acid (DCA), can induce reactive oxygen species (ROS) production, leading to oxidative DNA damage, genomic instability, and accumulation of oncogenic alterations (21, 22, 40). In parallel, bile acids can activate oncogenic signaling pathways, including β-catenin and c-Myc, thereby promoting tumor-cell proliferation, survival, and malignant progression.

Tryptophan metabolites, largely through AhR-dependent signaling, further connect metabolic reprogramming with immune regulation. Recent studies suggest that microbial and tumor-associated tryptophan metabolism influences metabolic competition between tumor cells and immune cells, alters nutrient availability, and contributes to the dysfunctional state of effector immune populations within the TME (19, 28, 44, 45). These effects link amino acid metabolism to both tumor-cell plasticity and immune escape.

Collectively, the metabolism–epigenetics axis represents a central interface through which microbial metabolites integrate transcriptional regulation, cellular metabolism, and immunological function in gastrointestinal cancer.

### 2.4 Bidirectional and context-dependent effects

A critical feature of gut microbiota-derived metabolites is that their biological functions are highly context-dependent and frequently bidirectional. Their effects are shaped by multiple variables, including metabolite concentration, tissue distribution, tumor stage, host metabolic status, microbial ecology, and the immune composition of the tumor microenvironment (4, 5, 8, 10, 17, 21, 22, 28).

For example, SCFAs are generally regarded as anti-inflammatory and tumor-suppressive metabolites because they reinforce epithelial barrier integrity, inhibit HDAC activity, and promote anti-tumor immune responses. However, under specific conditions, particularly in immunoregulatory settings, SCFAs may also promote regulatory T-cell expansion and contribute to immune tolerance (13, 20, 23, 34, 48). Likewise, bile acids maintain metabolic and immune homeostasis under physiological conditions, but under pathological conditions—such as high-fat diet exposure, dysbiosis, or chronic inflammation—secondary bile acids can promote epithelial injury, oxidative stress, immune suppression, and tumor progression (10, 17, 21, 22, 27, 40).

Tryptophan metabolites also exhibit dual and context-dependent roles. Under physiological conditions, balanced AhR activation by microbial indole derivatives contributes to mucosal homeostasis, epithelial defense, and controlled immune regulation. In contrast, persistent activation of the kynurenine–AhR axis within the tumor microenvironment can drive immunosuppression, T-cell dysfunction, and tumor progression (8, 12, 18, 19, 24, 28, 49).

These observations indicate that gut microbial metabolites should not be classified simply as tumor-promoting or tumor-suppressive. Rather, their biological effects depend on ligand specificity, receptor repertoire, cellular context, and microenvironmental conditions, underscoring the need for a dynamic and systems-level framework to understand metabolite-dependent regulation in gastrointestinal cancer (4, 5, 8, 28).

Importantly, context dependency is not merely a conceptual framework but can be experimentally quantified. For SCFAs, local concentrations, receptor expression patterns (FFAR2, FFAR3, and HCAR2), and immune-cell composition influence whether regulatory or cytotoxic immune programs predominate (23, 35, 37, 50). For bile acids, the ratio of primary to secondary bile acids and the abundance of microbial bile-acid–transforming taxa can be assessed using targeted metabolomics and metagenomic profiling (21, 22, 41, 42, 51). Similarly, for tryptophan metabolites, AhR ligand availability, indoleamine 2,3-dioxygenase 1 (IDO1)/tryptophan 2,3-dioxygenase (TDO) activity, and local kynurenine-to-tryptophan ratios may provide functional indicators of pathway activation (18, 19, 24, 49). These measurable variables may help define biologically meaningful thresholds that distinguish tumor-suppressive from tumor-promoting metabolite activity.

## 3 Major classes of gut microbiota-derived metabolites in gastrointestinal cancer

Based on their sources, biochemical properties, and functional roles in tumor biology, gut microbiota-derived metabolites involved in gastrointestinal cancer can be broadly categorized into three major classes: SCFAs, bile acids, and tryptophan metabolites. Although these metabolite groups differ in origin and signaling properties, they exhibit substantial functional overlap and crosstalk in shaping immune responses, epithelial homeostasis, metabolic reprogramming, and tumor progression. Although the strongest mechanistic evidence is currently derived from colorectal cancer models, emerging studies indicate that microbial-metabolite pathways also influence gastric, esophageal, hepatobiliary, and pancreatic malignancies through tumor-type-specific immune and metabolic mechanisms (52, 53). Representative SCFAs and bile acids, together with their major microbial producers, immunological functions, and biological variability across tumor-related contexts, are summarized in **Table 1**.

**Table 1:** Major gut microbiota-derived SCFAs and bile acids: representative microbial producers, immunological functions, and relevance to gastrointestinal cancer.

Metabolite	Representative producing taxa / microbial source	Major mechanisms and effects on TME/immune regulation	Context-dependent features	Representative references
Butyrate	*Faecalibacterium prausnitzii*, *Roseburia* spp., *Eubacterium rectale*	HDAC inhibition; activation of HCAR2 (GPR109A) and FFAR3; promotes Tregs differentiation, enhances CD8^+^ T cells metabolic fitness, suppresses NF-κB signaling, maintains epithelial barrier integrity	Generally protective at physiological concentrations; effects vary with concentration, receptor expression, immune context, and tumor stage	(13, 20, 23, 26, 34, 48, 50)
Propionate	*Bacteroides* spp., *Akkermansia muciniphila*, *Veillonella* spp.	Activates FFAR2/FFAR3; regulates cytokine production, Tregs differentiation, and epithelial homeostasis	Effects depend on local concentration, receptor distribution, and microbial composition	(20, 23, 26, 34)
Acetate	*Bifidobacterium* spp., *Akkermansia muciniphila*, acetogenic bacteria	Activates FFAR2; supports epithelial energy metabolism, barrier maintenance, and immune signaling	Usually protective but may exert different effects under dysbiosis and chronic inflammation	(20, 23, 26, 35)
Deoxycholic acid (DCA)	Produced from primary bile acids by 7α-dehydroxylating bacteria including *Clostridium scindens* and *Clostridium hylemonae*	Induces ROS production, DNA damage, NF-κB/STAT3 activation, and inflammatory signaling; modulates immune-cell populations	High concentrations promote carcinogenesis; effects depend on bile-acid composition and microbial transformation capacity	(17, 21, 22, 27, 40)
Lithocholic acid (LCA)	Produced from chenodeoxycholic acid by 7α-dehydroxylating *Clostridium* spp.	Regulates oxidative stress, immune signaling, and epithelial responses; precursor of several immunoregulatory bile-acid derivatives	Parent LCA may be pro-tumorigenic, whereas certain derivatives exhibit immunoregulatory activity	(21, 22, 40, 51, 60, 61)
Ursodeoxycholic acid (UDCA)	Formed through microbial epimerization of primary bile acids	Reduces bile acid–induced epithelial injury, oxidative stress, and inflammation; investigated as a chemopreventive bile acid	Protective effects vary according to disease state and bile-acid pool composition	(21, 22, 40)
3-oxoLCA	Microbiota-modified LCA derivative	Directly inhibits TH17 cells differentiation through modulation of RORγt activity	Effects depend on microbial bile-acid transformation and local immune environment	(51)
isoalloLCA	Microbiota-modified LCA derivative	Promotes Foxp3 expression and regulatory T-cell differentiation	Structure-specific immunoregulatory activity	(51)
Microbiota-modified bile acids regulating RORγt^+^ Tregs	Secondary bile acids generated through microbial bile-acid metabolism	Maintain colonic RORγt^+^ regulatory T-cell homeostasis and mucosal immune balance	Dependent on microbial bile-acid–transforming capacity and intestinal immune status	(62)

SCFAs, short-chain fatty acids; TME, tumor microenvironment; DCA, deoxycholic acid; LCA, lithocholic acid; UDCA, ursodeoxycholic acid; HDAC, histone deacetylase; HCAR2, hydroxycarboxylic acid receptor 2; GPR109A, G protein-coupled receptor 109A; FFAR2, free fatty acid receptor 2; FFAR3, free fatty acid receptor 3; ROS, reactive oxygen species; NF-κB, nuclear factor-kappa B; STAT3, signal transducer and activator of transcription 3; Tregs, regulatory T cells; TH17, T helper 17. Representative producing taxa are listed as major microbial producers or transformation-associated organisms and do not represent an exhaustive list; RORγt, retinoic acid receptor-related orphan receptor gammat.

### 3.1 Short-chain fatty acids (SCFAs)

SCFAs, primarily acetate, propionate, and butyrate, are major metabolites generated by the gut microbiota through the fermentation of dietary fiber. As key signaling molecules at the microbiota–host interface, SCFAs play central roles in maintaining intestinal homeostasis and modulating gastrointestinal tumorigenesis (1, 2, 7, 20, 23, 26).

Beyond their function as metabolic substrates, SCFAs serve as pleiotropic signaling molecules that regulate immune responses, epithelial barrier integrity, and cellular metabolism. Through these coordinated activities, they influence gastrointestinal cancer development across multiple regulatory axes (4, 5, 13, 20, 23, 26). Emerging evidence further indicates that SCFAs act as important modulators of the tumor microenvironment by integrating metabolic and immune signaling in a highly dynamic and context-dependent manner (4, 5, 13, 26).

#### 3.1.1 SCFAs in inflammation–immunity regulation

Chronic inflammation is a major driver of colorectal carcinogenesis, and SCFAs play pivotal roles in modulating both innate and adaptive immune responses within the tumor microenvironment (7, 13, 20, 23).

At the molecular level, SCFAs exert their immunomodulatory effects through both G protein-coupled receptor signaling and HDAC inhibition. The major SCFA-responsive receptors include free fatty acid receptor 2 (FFAR2/GPR43), free fatty acid receptor 3 (FFAR3/GPR41), and hydroxycarboxylic acid receptor 2 (HCAR2/GPR109A), but these receptors differ in ligand preference and tissue distribution. Acetate preferentially activates FFAR2, propionate acts as a relatively balanced FFAR2/FFAR3 agonist, whereas butyrate can activate HCAR2 and FFAR3, particularly at relatively high local concentrations. These receptor-specific differences help explain why SCFAs produce distinct immune effects across epithelial, myeloid, and lymphoid compartments (20, 23, 35, 37).

In adaptive immunity, SCFAs can promote the differentiation and expansion of Tregs through upregulation of Foxp3 expression, thereby contributing to immune tolerance under homeostatic conditions (34, 48). At the same time, SCFAs can enhance the metabolic fitness and effector functions of CD8^+^ T cells by supporting mitochondrial metabolism and oxidative phosphorylation, thereby sustaining anti-tumor immunity in appropriate contexts (13, 29).

In innate immunity, SCFAs modulate macrophage polarization and dampen excessive inflammatory activation, thereby limiting inflammation-driven tumor promotion (54, 55). They also affect dendritic-cell activation and cytokine production, contributing to immune homeostasis and shaping the balance between tolerance and anti-tumor immunity (7, 23).

At the signaling level, SCFAs suppress NF-κB activation and reduce the production of pro-inflammatory mediators such as TNF-α and IL-6, thereby attenuating chronic inflammation-associated tumorigenesis (13, 20, 23).

Taken together, SCFAs coordinate inflammation control, immune-cell differentiation, and anti-tumor immune competence through receptor-dependent and HDAC-dependent mechanisms, with biological outcomes shaped by ligand abundance, receptor distribution, and immune-cell state.

#### 3.1.2 SCFAs and intestinal barrier integrity

Disruption of the intestinal barrier facilitates microbial translocation, persistent immune activation, and chronic inflammation, all of which contribute to colorectal carcinogenesis. SCFAs play fundamental roles in maintaining and restoring epithelial barrier function (2, 4, 5, 10, 20, 23).

Mechanistically, SCFAs enhance the expression and organization of tight junction proteins, including occludin, claudins, and zonula occludens-1 (ZO-1), thereby reducing intestinal permeability and reinforcing epithelial integrity (34–39, 56). SCFAs also promote epithelial-cell proliferation, differentiation, and mucosal renewal, which are essential for sustaining barrier homeostasis (39, 56, 57).

In addition to structural maintenance, SCFAs facilitate epithelial repair and resilience to injury. They activate AMPK-related signaling, induce autophagy, and support epithelial metabolic adaptation, thereby promoting mucosal healing and barrier recovery under inflammatory conditions (26, 38, 39, 57).

Emerging evidence further suggests that SCFAs indirectly contribute to barrier stability by modulating microbial composition, favoring beneficial commensals and restraining pro-inflammatory or pathogenic taxa. Through these direct and indirect actions, SCFAs help establish a protective microbiota–metabolite–barrier feedback loop that limits inflammation-associated tumor promotion (2, 4, 5, 7, 26).

#### 3.1.3 SCFAs in metabolism and epigenetic regulation

SCFAs, particularly butyrate, are key epigenetic regulators that influence tumor-cell behavior through inhibition of HDACs. By altering chromatin accessibility and transcriptional programs, SCFAs regulate cell proliferation, differentiation, and apoptosis in gastrointestinal cancer cells (20, 26, 58).

At the level of cell-cycle control, butyrate can upregulate cyclin-dependent kinase inhibitors such as p21 and p27, thereby inducing cell-cycle arrest and suppressing tumor-cell proliferation (26, 58). SCFAs can also promote apoptosis through modulation of Bcl-2 family signaling and activation of caspase-dependent pathways, further contributing to tumor suppression (20, 26, 46, 47).

Beyond epigenetic regulation, SCFAs influence tumor-associated metabolism. They can modulate glycolytic flux, mitochondrial activity, and nutrient utilization, thereby counteracting metabolic programs that favor malignant progression (26, 44–47). These effects are closely linked to cancer metabolic reprogramming and to the metabolic state of the tumor microenvironment.

Recent studies further suggest that SCFAs shape metabolic interactions between tumor cells and immune cells within the tumor microenvironment. By influencing immune-cell metabolism, mitochondrial fitness, and nutrient competition, SCFAs may affect the balance between anti-tumor immunity and tumor growth at a systems level (4, 5, 13, 29, 44, 45).

Collectively, these findings indicate that SCFAs function not only as microbial fermentation products but also as epigenetic and metabolic regulators that link microbial activity to tumor-cell fate and tumor microenvironmental remodeling.

### 3.2 Bile acids

Bile acids are cholesterol-derived metabolites synthesized in the liver and subsequently modified by the gut microbiota through deconjugation, dehydroxylation, and other biotransformation reactions. Beyond their classical functions in lipid digestion and absorption, bile acids are now recognized as important signaling molecules that regulate host metabolism, mucosal immunity, epithelial homeostasis, and tumor progression (10, 17, 19, 21, 22).

Within the intestine, primary bile acids are converted by microbial enzymes into secondary bile acids, including deoxycholic acid (DCA) and lithocholic acid (LCA). These transformations markedly alter their physicochemical properties and biological activities, thereby influencing epithelial integrity, inflammatory signaling, immune regulation, and carcinogenesis (4, 5, 21, 22). Emerging evidence suggests that dysregulated bile acid metabolism constitutes a critical mechanistic link between gut microbial imbalance and gastrointestinal tumorigenesis (5, 10, 21, 22, 41, 42).

Overall, the biological effects of bile acids are determined by bile-acid species, local concentration, receptor engagement, and microbial transformation. Under physiological conditions, they contribute to metabolic and immune homeostasis; however, under pathological conditions—particularly when secondary bile acids accumulate—their tumor-promoting effects often predominate through coordinated regulation of inflammation, barrier disruption, oxidative stress, and immune suppression (10, 21, 22, 27, 40).

Beyond colorectal cancer, bile acid–microbiota interactions are particularly relevant to hepatobiliary malignancies because bile acids circulate through the enterohepatic axis and directly shape hepatic immune surveillance (59). In liver cancer models, gut microbiome-dependent bile acid metabolism has been shown to regulate hepatic CXCL16 expression and the accumulation of CXCR6^+^ natural killer T cells, thereby linking microbial bile acid transformation to liver-selective antitumor immunity (52). These findings suggest that bile acid signaling may connect intestinal dysbiosis with hepatocellular carcinoma and cholangiocarcinoma through immune-mediated mechanisms.

#### 3.2.1 Bile acids in inflammation and immune regulation

Bile acids regulate immune responses primarily through activation of nuclear and membrane-associated receptors, including the farnesoid X receptor (FXR) and other bile acid-responsive signaling pathways, as well as through modulation of inflammatory cascades (10, 21, 22, 60, 61).

In addition to FXR-related signaling, specific microbiota-modified bile acid derivatives directly regulate intestinal T-cell differentiation. For example, 3-oxoLCA inhibits TH17 cells differentiation, whereas isoalloLCA promotes regulatory T-cell differentiation, illustrating how structurally distinct bile acid metabolites can exert divergent immunological effects (51). Furthermore, microbial bile acid metabolites contribute to the maintenance of colonic RORγt^+^ regulatory T-cell homeostasis, highlighting bile acids as important regulators of mucosal immune balance (62).

Under physiological conditions, FXR signaling contributes to intestinal immune homeostasis by restraining excessive inflammatory responses and maintaining mucosal metabolic balance, thereby exerting protective effects against early tumor-promoting inflammation (21, 22, 60, 61). In contrast, under conditions of bile acid dysregulation—such as high-fat diet exposure, microbial imbalance, or chronic inflammatory stress—secondary bile acids accumulate and promote pro-inflammatory signaling, including activation of NF-κB and increased production of cytokines such as TNF-α and IL-6, thereby favoring chronic inflammation and tumorigenesis (21, 22, 27, 40).

Importantly, bile acids also exert substantial effects on anti-tumor immunity. Recent studies indicate that microbiota-modified bile acids can directly suppress CD8^+^ T-cell effector functions, reduce cytotoxic activity, and impair immune surveillance, thereby facilitating colorectal cancer progression (27). In addition, bile acids can influence macrophage polarization and promote tumor-associated macrophage (TAM)-like phenotypes, further reinforcing an immunosuppressive tumor microenvironment (11, 21, 22).

Taken together, these findings indicate that bile acids promote tumor progression through a dual mechanism involving inflammatory activation and immune suppression, making them central mediators of tumor–immune regulation in gastrointestinal cancer.

#### 3.2.2 Bile acids and intestinal barrier disruption

Disruption of intestinal barrier integrity is a key mechanism through which dysregulated bile acid metabolism promotes gastrointestinal tumorigenesis. Excessive accumulation of secondary bile acids exerts direct cytotoxic effects on epithelial cells, perturbs tight junction architecture, and increases intestinal permeability, thereby facilitating microbial translocation and chronic mucosal inflammation (10, 21, 22, 40).

Barrier dysfunction further promotes the entry of bacteria and microbial products into the lamina propria and systemic compartment, thereby sustaining inflammatory signaling that favors tumor initiation and progression (10, 20, 22, 43). In parallel, bile acids reshape gut microbial composition, promoting dysbiosis characterized by enrichment of pro-inflammatory or pro-tumorigenic taxa and depletion of beneficial commensals (21, 22, 41, 42).

Taken together, these observations support a pathogenic cascade of bile acid dysregulation, microbiota imbalance, barrier disruption, and chronic inflammation. This integrated “microbiota dysbiosis–barrier disruption–inflammation” axis appears to be an important mechanism linking bile acid metabolism to colorectal carcinogenesis (4, 5, 22, 43).

#### 3.2.3 Bile acids in oxidative stress and genomic instability

Secondary bile acids, particularly deoxycholic acid (DCA) and lithocholic acid (LCA), induce oxidative stress and contribute to genomic instability in gastrointestinal epithelial cells. By generating ROS and promoting DNA damage, they increase the risk of oncogenic mutations and chromosomal alterations that drive colorectal carcinogenesis (21, 22, 27, 40–42).

ROS generated by secondary bile acids also activate multiple oncogenic signaling pathways, including NF-κB, STAT3, and β-catenin, which promote cell survival, proliferation, and malignant progression (21, 22, 27, 40–43). Furthermore, oxidative stress can exacerbate inflammation and compromise DNA repair mechanisms, creating a microenvironment conducive to tumor initiation and progression (10, 22, 27, 40).

In addition to direct epithelial effects, secondary bile acids contribute indirectly to genomic instability by altering the gut microbial community, enhancing dysbiosis-associated metabolic and inflammatory stress, and amplifying DNA-damaging signals (19, 22, 41, 42). This combination of direct cytotoxicity, oxidative stress, and microbial-mediated inflammation forms a multifactorial mechanism by which bile acid dysregulation promotes colorectal carcinogenesis.

### 3.3 Tryptophan metabolites

Tryptophan metabolites, derived from host and microbial metabolism, include indole derivatives, kynurenine, and serotonin. These metabolites act as signaling molecules that influence immune modulation, epithelial barrier function, metabolic reprogramming, and tumor progression in gastrointestinal cancer (12, 18, 19, 24, 28).

Although most mechanistic studies of tryptophan metabolism in gastrointestinal cancer have focused on colorectal cancer, tryptophan–AhR signaling may also be relevant to upper gastrointestinal and pancreatic malignancies. In gastric cancer, *Helicobacter pylori* infection and gastric dysbiosis remodel the tumor immune microenvironment and may interact with microbial metabolic pathways that regulate inflammation and immune escape (63). In esophageal squamous-cell carcinoma, oral and esophageal microbiota, including *Streptococcus anginosus* (64), have been associated with carcinogenesis and treatment response (65), supporting a broader role for microbiota–immune interactions beyond the colon. In pancreatic ductal adenocarcinoma, tumor-associated microbiota can promote oncogenesis through innate and adaptive immune suppression, suggesting that microbial-metabolite pathways may influence pancreatic tumor immunity (53).

Microbial tryptophan catabolism generates indole derivatives that activate the AhR in epithelial and immune cells, modulating transcriptional programs involved in mucosal immunity and epithelial homeostasis (12, 18, 19, 24, 28). Kynurenine, produced via host and tumor-associated IDO1 activity, also engages AhR signaling, linking tryptophan metabolism to immune tolerance and tumor immune evasion (12, 19, 24, 28).

The biological effects of tryptophan metabolites depend on ligand abundance, AhR signaling status, tissue environment, and microbial composition. While physiological levels support epithelial integrity and controlled immune regulation, dysregulated tryptophan metabolism contributes to immunosuppression, T-cell dysfunction, and tumor progression (8, 12, 18, 19, 24, 28).

#### 3.3.1 Tryptophan metabolites in inflammation–immunity regulation

Tryptophan-derived indole metabolites and kynurenine modulate both innate and adaptive immunity in the gastrointestinal tract (12, 18, 19, 24).

Indole derivatives act primarily through AhR activation in epithelial and immune cells, promoting regulatory T-cell differentiation, limiting excessive inflammatory signaling, and supporting mucosal tolerance (12, 18, 24).

Kynurenine produced by IDO activity similarly activates AhR in T cells and myeloid populations, enhancing expansion of Tregs and MDSCs, thereby suppressing anti-tumor immunity and promoting immune evasion (12, 19, 24, 28).

These metabolites also modulate dendritic cell maturation and cytokine production, contributing to the establishment of an immunosuppressive tumor microenvironment while maintaining mucosal homeostasis under physiological conditions (12, 19, 24).

#### 3.3.2 Tryptophan metabolites and the barrier–microbiota axis

Tryptophan metabolites contribute to intestinal barrier integrity and homeostasis by modulating epithelial function and interacting with the gut microbiota. Microbial indole derivatives activate AhR in epithelial cells, promoting tight junction protein expression and mucosal regeneration, thereby reducing intestinal permeability and limiting microbial translocation (12, 18, 19, 24, 28, 43).

Dysregulation of tryptophan metabolism—such as excessive kynurenine production—can compromise barrier function, increase epithelial susceptibility to injury, and enhance inflammatory signaling, contributing to tumorigenesis (12, 18, 19, 24, 28). These effects are further amplified by interactions with the microbiota, which influence tryptophan availability and metabolite profiles, establishing a bidirectional “tryptophan–microbiota–barrier” regulatory loop (18, 19, 28, 43).

Collectively, these mechanisms highlight the integral role of tryptophan metabolites in maintaining mucosal barrier integrity, coordinating host–microbe interactions, and modulating inflammation-associated tumor risk in the gastrointestinal tract.

#### 3.3.3 Tryptophan metabolites in tumor cell behavior and metabolic reprogramming

Tryptophan metabolism influences tumor cell behavior and the metabolic landscape of the tumor microenvironment through multiple mechanisms. Kynurenine, produced via IDO1/TDO activity, acts as an immunosuppressive and metabolic regulator by activating AhR in both tumor and immune cells, thereby promoting tumor-cell survival, proliferation, and immune evasion (12, 19, 24, 28).

Indole derivatives generated by microbial metabolism can also influence tumor cells by modulating epithelial differentiation, apoptosis, and signaling pathways involved in cellular metabolism, including glycolysis and mitochondrial function (18, 19, 24, 45). These metabolites therefore link microbial activity to tumor metabolic reprogramming and the regulation of nutrient competition within the tumor microenvironment.

Emerging evidence indicates that dysregulated tryptophan metabolism facilitates metabolic crosstalk between tumor cells and immune cells. Kynurenine-mediated AhR activation can impair CD8^+^ T-cell effector function, modulate regulatory T-cell activity, and alter myeloid cell metabolism, ultimately creating a metabolically permissive environment for tumor growth (4, 12, 19, 24, 28, 44, 45).

Collectively, these findings demonstrate that tryptophan metabolites serve as key modulators of tumor-cell fate and metabolic adaptation, integrating epigenetic, immunologic, and metabolic cues to shape the gastrointestinal tumor microenvironment.

## 4 Immune modulation and the tryptophan–AhR axis

The TME is a dynamic ecosystem where tumor cells, immune populations, and metabolic factors interact to regulate tumor progression and therapeutic response. Among these regulatory pathways, the tryptophan–AhR axis has emerged as a central mechanism linking host- and microbiota-derived tryptophan metabolites to immune modulation in gastrointestinal cancers (12, 18, 24, 28).

Tryptophan metabolism generates kynurenine through IDO1/TDO activity and various microbial indole derivatives, which act as endogenous ligands for AhR (12, 18, 24, 28). AhR activation orchestrates a coordinated immunoregulatory network by modulating Tregs, MDSCs, CD8^+^ T-cell function, and cytokine production, while also integrating environmental cues such as hypoxia and inflammatory signals (24, 28, 66, 67).

This network exhibits context-sensitive immunometabolic regulation: under physiological conditions, tryptophan metabolites maintain immune tolerance, barrier integrity, and mucosal homeostasis, whereas under dysregulated conditions—such as elevated kynurenine levels, microbial dysbiosis, or chronic inflammation—they promote immunosuppression, CD8^+^ T-cell exhaustion, Tregs expansion, and tumor progression. The following subsections (4.1–4.6) elaborate these mechanisms in detail, highlighting AhR as a central hub coordinating metabolic and immune interactions within the TME (12, 18, 24, 28, 49).

### 4.1 AhR signaling as a central regulatory hub

AhR is a ligand-activated transcription factor that senses environmental, dietary, microbial, and metabolic cues. In its inactive state, AhR is retained in the cytoplasm as part of a multiprotein complex. Upon ligand binding, AhR translocates to the nucleus, heterodimerizes with the AhR nuclear translocator (ARNT), and binds dioxin/xenobiotic response elements (DREs/XREs) to regulate transcriptional programs involved in immune tolerance, inflammation, barrier function, and cellular differentiation (12, 18, 24, 68).

Importantly, AhR signaling is not uniform across ligands or cell types. Different tryptophan-derived metabolites may generate ligand-selective transcriptional responses, and AhR expression varies across epithelial, lymphoid, myeloid, and tumor-associated cell populations, contributing to tissue-specific outcomes within the TME (49, 69).

AhR signaling is also subject to negative-feedback regulation. One important mechanism is induction of the AhR repressor (AhRR), which competes with AhR for ARNT binding and limits excessive or prolonged AhR activation. This feedback loop may help maintain mucosal immune homeostasis under physiological conditions, whereas persistent or dysregulated AhR activation in cancer can shift immune responses toward tolerogenic and immunosuppressive states (68).

By integrating microbial, tumor-derived, hypoxic, and inflammatory signals, AhR functions as a central regulatory hub coordinating metabolic and immune interactions within the TME. This mechanistic complexity supports the central conceptual role of the tryptophan–AhR axis but also indicates that therapeutic targeting should consider ligand specificity, receptor availability, and cell-type-specific signaling rather than treating AhR activation as a single uniform pathway (12, 18, 24, 28).

### 4.2 CD8^+^ T cell dysfunction and exhaustion

CD8^+^ T cells are primary effectors of anti-tumor immunity, but in the TME they often become exhausted. Activation of the IDO1–kynurenine–AhR axis suppresses CD8^+^ T-cell activity by upregulating inhibitory receptors PD-1, CTLA-4, and Tim-3 (24, 28, 66).

Exhausted CD8^+^ T cells exhibit reduced production of IFN-γ, granzyme B, and perforin, impairing cytotoxicity. AhR signaling also alters mitochondrial metabolism, further compromising effector function (24, 44, 66, 70). These mechanisms collectively attenuate anti-tumor immunity in gastrointestinal cancers.

### 4.3 Tregs expansion and immune tolerance

Tregs maintain immune homeostasis but their expansion in tumors contributes to immunosuppression.

AhR activation promotes Tregs differentiation by upregulating Foxp3 and stabilizing the Tregs phenotype. Elevated kynurenine in the TME drives Tregs expansion, suppressing effector T-cell responses and enhancing immune tolerance (4, 18, 24, 28).

Tregs secrete immunosuppressive cytokines such as IL-10 and TGF-β, inhibiting CD8^+^ T cells activation and proliferation, establishing a feedback loop that reinforces an immunosuppressive microenvironment (4, 18, 24, 28). Together, these findings indicate that the kynurenine–AhR axis not only promotes Tregs expansion but also stabilizes immunosuppressive feedback loops that limit durable anti-tumor T-cell activity.

### 4.4 Metabolic reprogramming of immune cells

Tryptophan–AhR signaling alters immune cell metabolism to support immunosuppressive phenotypes. Kynurenine-mediated AhR activation shifts T-cell metabolism toward reduced mitochondrial respiration and glycolysis, limiting effector function, while promoting metabolic fitness of Tregs and MDSCs (24, 44, 45). These changes may further exacerbate immunometabolic competition within the TME, thereby favoring tumor persistence over effective immune clearance.

### 4.5 Cytokine production and immune modulation

AhR activation regulates the production of immunomodulatory cytokines. Kynurenine enhances IL-10 and TGF-β, suppressing effector T-cell activity, whereas indole derivatives can promote IL-22 and mucosal protective cytokines, maintaining barrier integrity and immune homeostasis (12, 24, 25, 71). Accordingly, the net immunologic outcome of AhR signaling depends on ligand source, metabolite abundance, and the inflammatory context of the local microenvironment.

### 4.6 Therapeutic implications

Targeting the tryptophan–AhR axis presents opportunities for cancer therapy. Strategies include IDO1/TDO inhibitors, modulation of microbial tryptophan metabolism, and AhR antagonists, with the aim of restoring anti-tumor immunity and reducing Tregs/MDSC-mediated immunosuppression (24, 28, 72, 73). However, the limited efficacy observed in some clinical programs indicates that inhibition of a single upstream enzyme may be insufficient in the context of pathway redundancy, compensatory immunosuppressive circuits, and persistent downstream AhR activation (72–74). These findings support a broader pathway-level strategy that integrates upstream metabolic targeting with downstream immune reprogramming and rational combination therapy (74).

Representative tryptophan-derived metabolites, their major microbial producers, signaling pathways, and immunological functions are summarized in **Table 2**.

**Table 2:** Major tryptophan-derived metabolites: representative microbial producers, signaling pathways, and immunological functions relevant to gastrointestinal cancer.

Metabolite	Representative producing taxa / source	Major receptor or pathway	Principal immunological and biological functions	Relevance to gastrointestinal cancer	Representative references
Kynurenine (Kyn)	Host IDO1/TDO-mediated tryptophan metabolism	AhR	Promotes Tregs differentiation, MDSC activation, CD8^+^ T-cell dysfunction, and immune tolerance	Frequently associated with tumor immune escape and poor prognosis	(12, 18, 24, 28, 49)
Kynurenic acid (KYNA)	Host kynurenine pathway	AhR, GPR35	Immunomodulation, anti-inflammatory signaling, regulation of immune-cell activity	Emerging regulator of tumor-associated immune responses	(18, 24, 49)
Quinolinic acid (QA)	Host kynurenine pathway	NAD^+^ biosynthesis pathway	Immunometabolic regulation, oxidative stress, and inflammatory signaling	May contribute to tumor-associated metabolic remodeling	(18, 24)
Picolinic acid (PA)	Host kynurenine pathway	Metal-ion chelation and immune modulation pathways	Regulates macrophage function and antimicrobial responses	Potential role in immune regulation remains under investigation	(18, 24)
Indole	*Escherichia coli*, *Bacteroides* spp., *Clostridium* spp.	AhR	Supports mucosal immune homeostasis and epithelial barrier function	Protective effects on intestinal homeostasis	(24, 25, 28)
Indole-3-acetic acid (IAA)	*Bacteroides* spp., *Clostridium* spp., *Peptostreptococcus* spp.	AhR	Anti-inflammatory signaling, regulation of cytokine production, maintenance of epithelial integrity	Associated with improved mucosal homeostasis and immune regulation	(14, 24, 25)
Indole-3-propionic acid (IPA)	*Clostridium sporogenes*, *Peptostreptococcus* spp.	AhR, pregnane X receptor (PXR)	Enhances epithelial barrier integrity, antioxidant activity, and immune homeostasis	Potential anti-tumor and barrier-protective effects	(24, 25)
Indole-3-aldehyde (IAld)	*Lactobacillus reuteri* and related lactobacilli	AhR	Induces IL-22 production, promotes mucosal defense, and supports epithelial barrier maintenance	Important mediator of microbiota–immune communication	(24, 25)
Indole-3-lactic acid (ILA)	*Lactobacillus* spp., *Bifidobacterium* spp.	AhR	Regulates immune tolerance and epithelial homeostasis	Emerging immunoregulatory metabolite	(24, 25)
Tryptamine	*Clostridium sporogenes*, *Ruminococcus gnavus*	Serotonin-related signaling, GPCR pathways	Modulates intestinal motility, epithelial responses, and immune signaling	Potential indirect effects on tumor-associated microenvironments	(24, 25)
Microbial indole derivatives (general)	*Lactobacillus*, *Bifidobacterium*, *Bacteroides*, *Clostridium* spp.	AhR	Promote epithelial barrier integrity, immune homeostasis, and balanced inflammatory responses	Generally associated with anti-inflammatory and protective effects	(24, 25, 28)

AhR, aryl hydrocarbon receptor; IDO1, indoleamine 2,3-dioxygenase 1; TDO, tryptophan 2,3-dioxygenase; Kyn, kynurenine; KYNA, kynurenic acid; QA, quinolinic acid; PA, picolinic acid; IAA, indole-3-acetic acid; IPA, indole-3-propionic acid; IAld, indole-3-aldehyde; ILA, indole-3-lactic acid; PXR, pregnane X receptor; GPR35, G protein-coupled receptor 35; GPCR, G protein-coupled receptor; Tregs, regulatory T cells; MDSCs, myeloid-derived suppressor cells. Representative producing taxa are listed as major microbial producers or pathway-associated sources and do not represent an exhaustive list; ARNT, AhR nuclear translocator.

## 5 Synthesis and outlook

The gut microbiota-derived metabolite network has emerged as a critical regulator of gastrointestinal tumorigenesis by integrating microbial activity with host immune responses, epithelial barrier integrity, and metabolic signaling (4, 18, 24). Rather than acting as isolated effectors, SCFAs, bile acids, and tryptophan-derived metabolites converge on overlapping immunometabolic pathways that shape the tumor microenvironment and therapeutic responsiveness (18, 24, 26, 45). This systems-level perspective helps explain why disturbances in different metabolite classes may ultimately produce similar tumor-promoting outcomes, including immune suppression, barrier dysfunction, and metabolic reprogramming (26, 44).

However, translating this framework into clinically useful strategies requires careful consideration of evidence strength, tumor-type specificity, ligand specificity, and host–microbiome variability (49, 69, 75). The following sections therefore synthesize unresolved mechanistic tensions, sources of inter-study variability, the central role of the tryptophan–AhR axis, and future opportunities for multi-omics-guided precision intervention.

### 5.1 From individual metabolites to integrated regulatory networks

Gastrointestinal tumorigenesis is regulated not by individual metabolites in isolation, but by an integrated network of microbial and host-derived metabolites that orchestrate immune, barrier, and metabolic processes within the TME (4, 18, 24, 26, 44).

SCFAs, including acetate, propionate, and butyrate, promote Tregs differentiation (34, 48), enhance CD8^+^ T cells cytotoxicity under homeostatic conditions (13, 50), and regulate cytokine production via HDAC inhibition (34–39) and GPCR signaling (20, 23, 35, 37). They also maintain epithelial barrier integrity through upregulation of tight junction proteins (ZO-1, occludin, claudins) (34–39, 56, 57). Dysregulated SCFA production, such as in microbial dysbiosis, shifts immune responses toward tumor-promoting phenotypes (26, 46).

Bile acids, particularly secondary forms like DCA and LCA, influence oxidative stress, DNA damage, and oncogenic signaling (NF-κB, STAT3, β-catenin) (21, 22, 27, 40), regulate immune cell populations, and modulate cytokine profiles (51, 62). Their biological effects are influenced by metabolite concentration, receptor expression, and microbial transformation, resulting in substantial variability across physiological and pathological settings (21, 22, 51, 62).

Tryptophan-derived metabolites, including kynurenine (IDO1/TDO-mediated) and microbial indole derivatives, activate AhR signaling to coordinate Tregs expansion, MDSC activity, CD8^+^ T-cell function, and barrier integrity (12, 18, 24, 28, 49). Kynurenine favors immunosuppressive phenotypes, whereas indoles maintain mucosal homeostasis, illustrating context-dependent duality.

The cross-talk among SCFAs, bile acids, and tryptophan metabolites establishes a multilayered regulatory network integrating immune modulation, barrier maintenance, oxidative stress control, and metabolic reprogramming. Perturbations such as microbial dysbiosis, chronic inflammation, or tumor-driven metabolic shifts drive the system toward tumor-promoting states (4, 18, 24, 26, 44). This systems-level view may help explain why perturbations in different metabolite classes often converge on similar tumor-promoting phenotypes.

A further limitation of the current literature is that both the distribution and strength of evidence vary substantially across gastrointestinal malignancies and metabolite classes. Mechanistic evidence is most extensive in colorectal cancer, whereas gastric, esophageal, hepatobiliary, and pancreatic cancers remain less well characterized (52, 53, 63, 65). This imbalance likely reflects differences in sample accessibility, tumor-associated microbial biomass, anatomical exposure to luminal microbes, and the availability of validated animal models. In addition, while several studies have demonstrated causal links through germ-free models, metabolite supplementation, microbial transfer, or receptor-specific interventions, a large proportion of the current literature remains based on associative observations from microbiome profiling or cross-sectional metabolomic analyses (52, 53). Future studies should therefore adopt tumor-type-specific designs and distinguish causal mechanisms from correlative metabolite–tumor associations rather than assuming that mechanisms defined in colorectal cancer can be directly generalized to all gastrointestinal malignancies.

### 5.2 Determinants of metabolite-specific biological responses

The biological effects of microbiota-derived metabolites are influenced by multiple interacting factors rather than by metabolite identity alone. Variability in metabolite concentration, receptor expression, tissue localization, microbial composition, and immune-cell state can substantially alter downstream biological responses, contributing to differences observed across experimental models and clinical studies.

SCFAs provide a representative example of this complexity. Butyrate promotes regulatory T-cell differentiation through HDAC inhibition and enhanced Foxp3 expression, yet it can also support CD8^+^ T cells metabolic fitness, memory formation, and antitumor activity. These seemingly divergent outcomes likely reflect differences in local metabolite concentration, cellular targets, and immune context rather than genuine biological inconsistency (50).

Similarly, the biological consequences of bile acid signaling depend not only on bile acid species but also on microbial transformation, receptor availability, and tissue distribution. Secondary bile acids such as DCA and LCA can promote oxidative stress, DNA damage, and oncogenic signaling under pathological conditions, whereas microbiota-modified bile acid derivatives may support immune homeostasis through regulation of Tregs and TH17 cells differentiation (51, 62).

For tryptophan metabolism, biological outcomes are shaped by the relative abundance of kynurenine-pathway metabolites and microbial indole derivatives, as well as by cell-type-specific AhR signaling. Kynurenine generally favors immunosuppressive phenotypes, whereas microbial indoles contribute to epithelial barrier maintenance and balanced immune responses. Emerging evidence further suggests that different AhR ligands exhibit distinct receptor-binding properties and transcriptional outputs, indicating that ligand-specific signaling may be an important determinant of biological function (49).

Collectively, these observations suggest that differences in metabolite concentration, receptor signaling, microbial ecology, and host immune status may underlie many of the apparently conflicting findings reported in the literature. Future studies should therefore move beyond simplified classifications of metabolites as either beneficial or harmful and instead focus on defining the biological conditions under which specific metabolite-driven responses emerge.

### 5.3 The tryptophan–AhR axis as a central immunoregulatory hub

The tryptophan–AhR axis acts as a central hub that integrates microbial metabolism, immune regulation, epithelial barrier function, and tumor-associated metabolic remodeling (12, 18, 24, 28, 49). However, this axis should not be interpreted as a simple linear kynurenine–AhR pathway. Although kynurenine is widely associated with AhR-dependent immunosuppression, recent evidence suggests that kynurenine-pathway derivatives and microbial indole metabolites differ substantially in AhR agonistic potency, ligand specificity, and downstream transcriptional outputs (76). Therefore, the immunological consequences of tryptophan metabolism depend not only on total kynurenine abundance but also on the repertoire of available AhR ligands, cell-type-specific AhR expression, and the inflammatory state of the tumor microenvironment (49).

Microbial indole derivatives often activate AhR-dependent programs that promote epithelial barrier maintenance, mucosal homeostasis, and balanced immune responses, whereas sustained activation of immunosuppressive tryptophan-catabolic pathways may favor Tregs expansion, MDSC activation, and CD8^+^ T-cell dysfunction. AhR also integrates environmental and metabolic cues, including HIF-1α- and NF-κB-related inflammatory signals, thereby linking hypoxia, inflammation, metabolism, and immune regulation within the TME. This ligand- and context-sensitive regulatory capacity distinguishes the tryptophan–AhR axis from other metabolite-associated pathways and supports its value as a conceptual and translational framework (12, 18, 24, 28).

### 5.4 Translational challenges and clinical implications

Patient-specific characteristics of the TME, including local metabolite concentrations, microbial composition, prior antibiotic exposure, and baseline immune contexture, must be considered when translating insights from the tryptophan–AhR axis into clinical interventions (49, 69, 75). The redundancy and compensatory capacity of metabolic pathways complicate therapeutic targeting, as blocking a single pathway may be insufficient to restore immune surveillance (44, 45, 70). Successful strategies are therefore likely to require integrated, multi-modal approaches that combine metabolic modulation, microbiome interventions, and immune-targeted therapies tailored to individual patient contexts (75, 77–80).

IDO1/TDO inhibitors have shown potential to reduce kynurenine levels, but clinical responses remain variable because of pathway redundancy, parallel immunosuppressive mechanisms, and persistent downstream AhR signaling (68, 72). The disappointing results of the phase III ECHO-301 trial further highlight the complexity of targeting tryptophan metabolism in cancer. Despite strong preclinical evidence supporting IDO1 inhibition, the combination of epacadostat and pembrolizumab failed to improve clinical outcomes in advanced melanoma. This discrepancy suggests that the immunological consequences of tryptophan metabolism may not be fully captured by inhibition of a single enzymatic step. Pathway redundancy, compensatory metabolic circuits, ligand-specific AhR signaling, and tumor-type-specific immune contexts may all contribute to therapeutic resistance. These findings emphasize the need for more precise patient stratification and biomarker-guided intervention strategies when targeting microbiota–metabolite–immune networks (81).

Additional translational barriers remain insufficiently addressed. For SCFA-based interventions, rapid absorption, limited colonic delivery, and substantial inter-individual variation in microbial production may complicate therapeutic implementation (75, 77, 82). Similarly, although the tryptophan–AhR axis has emerged as a promising target, reliable surrogate biomarkers of AhR activation *in vivo* remain poorly defined. Quantitative assessment of metabolite concentrations, receptor activity, downstream transcriptional signatures, and microbiome composition may therefore be necessary for patient stratification and treatment monitoring in future clinical studies (49, 77, 83).

In parallel, microbiome-based approaches, including diet-based modulation, microbial therapeutics, and microbiome-informed immunotherapy strategies, are increasingly supported by emerging clinical evidence (74, 77, 78, 84). These observations suggest that future translational designs should emphasize biomarker-guided patient selection, combination regimens, and context-specific host–microbiome profiling rather than single-pathway blockade alone (70, 72, 73, 75, 78, 84).

### 5.5 Future perspectives: toward multi-omics and precision medicine

Future research should leverage multi-omics integration combining microbiome composition, metabolomics, immune profiling, and host genomic context to define patient-specific TME characteristics more precisely (82, 85–89). Such integrative strategies may improve mechanistic resolution, identify actionable metabolite–immune interactions, and facilitate biomarker discovery for patient stratification and treatment-response prediction (75, 82, 85–88, 90). Precision medicine approaches may therefore include selective modulation of the microbiome, inhibition of the kynurenine–AhR axis, and personalized dietary or metabolite-targeted interventions designed around individual immune-metabolic states (77, 82, 84–86, 90, 91). Prospective longitudinal cohorts, adaptive biomarker-guided clinical trials, and standardized multi-center validation studies will be required to establish causal metabolite–immune relationships and facilitate clinical translation.

These strategies must account for the biological variability of microbial metabolites, as their effects are influenced by metabolite concentration, receptor availability, inflammatory status, tumor stage, and microenvironmental composition (71, 80, 87, 92). Integration of multi-omics data with clinical variables and computational tools may further support patient stratification and guide rational combination therapies targeting metabolic, microbial, and immune pathways simultaneously (75, 82, 85–88, 90). Advances in high-resolution metabolomics, single-cell immune profiling, microbiome engineering, and machine-learning-assisted multi-omics analysis are therefore likely to be essential for translating mechanistic insights into clinically actionable interventions (82, 86–88, 90).

## 6 Conclusion

Gut microbiota-derived metabolites have emerged as critical regulators of gastrointestinal tumorigenesis, serving as key intermediaries between microbial activity and host tumor biology (4, 7, 28, 30). Rather than exerting their effects through isolated signaling pathways, these metabolites participate in a dynamic and interconnected regulatory network that integrates immune modulation, intestinal barrier integrity, and metabolic–epigenetic reprogramming (4, 5, 26, 43).

Among them, SCFAs generally exert protective effects by preserving epithelial homeostasis and supporting anti-tumor immunity (13, 20, 23, 26, 48), whereas dysregulated bile acid metabolism and pathological tryptophan–AhR signaling more often contribute to tumor progression through inflammation, genomic instability, and immune suppression (22, 27, 40, 49, 59). Notably, the tryptophan–AhR axis serves as a central mechanistic hub that orchestrates immunosuppressive networks within the TME and provides a unifying framework for understanding tumor immune escape (18, 24, 28, 49, 69).

Despite these advances, the clinical effects of microbiota-derived metabolites are shaped by dynamic interactions among metabolite availability, receptor signaling, host immunity, and microbial ecology, contributing to substantial inter-individual variability and presenting major challenges for clinical translation. Future studies integrating multi-omics technologies, spatial and single-cell analyses, and longitudinal clinical validation will be essential for clarifying causal mechanisms and enabling precision therapeutic applications (30–32, 43, 88). A deeper understanding of the metabolite–immune–tumor network may not only refine current knowledge of gastrointestinal cancer biology but also facilitate the development of biomarker-driven and mechanistically informed therapeutic strategies (30–32, 75, 77).
